# Conflict of Interest in Nutrition: Where’s the Power? Comment on "Towards Preventing and Managing Conflict of Interest in Nutrition Policy? An Analysis of Submissions to a Consultation on a Draft WHO Tool"

**DOI:** 10.34172/ijhpm.2020.177

**Published:** 2020-09-30

**Authors:** Jody Harris, Nicholas Nisbett, Stuart Gillespie

**Affiliations:** ^1^Institute of Development Studies, University of Sussex, Brighton, UK; ^2^World Vegetable Center, Bangkok, Thailand.; ^3^International Food Policy Research Institute, Washington, DC, USA.

**Keywords:** Power, Conflict of Interest, Nutrition, Public Health, Private Sector, Accountability

## Abstract

Actual or perceived conflict of interests (COIs) among public and private actors in the field of nutrition must be managed. Ralston et al expose sharply contrasting views on the new World Health Organization (WHO) COI management tool, highlighting the contested nature of global debates. Both the WHO COI tool and the Ralston et al paper are largely quiet on aspects of power among different actors, however, which we argue is integral to these conflicts. We suggest that power needs to be acknowledged as a factor in COI; that it needs to be systematically assessed in COI tools using approaches we outline here; and that it needs to be explicitly addressed through COI mechanisms. We would recommend that all actors in the nutrition space (not only private companies) are held to the same COI standards, and we would welcome further studies such as Ralston et al to further build accountability.

## Acknowledging Power in Conflicts of Interest

The evolving nutrition transition,^1^ growing consensus on the commercial determinants of malnutrition,^2^ and accelerating processes of international trade^3^ are amplifying the potential for conflict of interest (COI) between private profit and public health nutrition issues globally. Despite a history of tension, governments and many non-governmental organisations and researchers have continued to engage with large companies to access funds, improve efficiency, and achieve scale and sustainability – though there is little evidence to date on when and how public-private partnerships work well for nutrition.^4^ Nonetheless, the prevailing ethos within global nutrition convenors such as the Scaling Up Nutrition (SUN) Movement has been for engagement with the private sector broadly defined^[1]^. Sustainable Development Goal target 17.17 explicitly aims to encourage and promote public-private partnerships^[2]^. We have argued before^[3]^ that COI issues emerging from the processes of changing food, health and social systems need to be debated openly, and the paper by Ralston and colleagues^5^ is a welcome empirical foray into this area.

The Ralston et al paper uses responses to a consultation on a new World Health Organization (WHO) COI approach as its data, finding strongly opposing positions between many member states, non-governmental organisations and academics broadly in support of the tool; and commercial companies, the SUN Movement, and the United States opposing it. The WHO tool^[4]^ itself is a 47-page document describing a 6-step process for government officials to follow in assessing potential COI at a national level. It contains suggested questionnaires asking for disclosure of funding sources and interests, and tables with guidance on what might constitute COI.

Formal definitions of COI in health, including that used by the WHO itself in these guidelines^[5]^, maintain that a COI is where a secondary interest (a vested interest in an alternative outcome) of another party might influence the primary mandate of a state institution to maintain and protect public health. This implies that there may be situations where *conflicting interests* (commercial/financial vs public health) do not necessarily lead to a *conflict of interest*, so long as the interests of the non-state actor are aligned with the state actor in that situation. We feel that, while this might make sense in the quasi-legalese of such WHO documents, in reality this implication is naïve to the relative power of different interest groups that shapes how COIs play out in practice. Ralston and colleagues’ research highlights the contested nature of the term ‘COI’ which in turn reflects conflicting interests and agendas.

Nutrition as a field is built upon the food system and closely aligned with health systems, both of which have been described as fields of power.^6,7^ In 2014, we highlighted nutrition as a similarly power-laden field.^8^ The issue of power is acknowledged in the introductory paper^[6]^ to the WHO guidelines, citing important work on the commercial determinants of non-communicable disease,^2^ but finds perfunctory treatment under ‘forms of engagement’ information in the draft WHO guidelines. Complementary to Ralston and colleagues’ research, therefore, and largely missing from the laudable initiatives and discussions at the WHO, is a systematic and nuanced attention to issues of power both in situations meeting the formal COI definition, and more broadly in the conflicting interests between public and private sectors in the context of public health nutrition.

## Assessing Power to Understand its Role in Conflicts of Interest

There is therefore a need to better understand how power plays out in different potential COI contexts. Much global nutrition debate relates to the power and interests of large companies, and this is our focus here as ‘Big Food’ is most likely to come into conflict with public health goals.^2^ Similarly, power and COI work at multiple scales, from global to local, and the international and national worlds are inextricably linked through processes of globalization and global development. Political and social science theory offers many different approaches to understanding and assessing power,^6,9,10^ but given the very practical nature of COI tools, we would like to highlight a practical and accessible approach called the Power Cube^[7]^. Building on traditional conceptualisations of power as ‘power to’ exert control, or ‘power over’ people or situations, the Power Cube looks for nuance in situations of power through assessing its different forms, spaces and levels.

This approach sees power as existing in different *forms*, from the most visible forms of influence through resources or social position, to invisible processes involving the internalisation of dominant norms and values clouding a person’s own view of her interests. A key form is hidden power, a type of action taken in the background of policy debates to exclude key issues from the agenda or create barriers to participation. Hidden power is highlighted, for example, in a recent critique of the International Life Sciences Institute (ILSI) in China,^11^ where this food industry lobby group was found to have influenced the narrative around national obesity policy. Through strong engagement in the country’s public health bodies, the ILSI was found to reinforce certain public health narratives over others, to the benefit of its member corporations – though the organisation has publicly denied these actions^[8]^.

The Power Cube also looks at different *spaces* in which power operates, including spaces into which only acceptable actors are invited to participate by authorities, and spaces which are claimed through citizen action by excluded groups. Many policy spaces remain closed to those whose interests are affected by those policies, with decisions made by certain actors behind closed doors, with little consultation or broader involvement. An example of closed spaces in the nutrition literature is analysis of how closed-door negotiations and opaque investor-state dispute settlement systems do not allow public health concerns to be adequately addressed in international trade agreements.^12^ This is particularly important given additional visible power imbalances between public health advocates and large corporations on the basis of access to manpower and resources, illustrating how these different facets of power interact.

While the examples above cite academic work on power in international nutrition, the Power Cube also offers an accessible framework for practical COI tools to incorporate assessments of power. Without an assessment of power, COI tools can only hope to understand and address some aspects of these conflicts. Understanding power may or may not allow those power asymmetries to be addressed, but will allow for them to be factored into COI assessments and actions – whether explicitly, in the guidance, or implicitly, in accepting that politics happens around these processes that formal COI guidelines can only partly address. Developing our understanding of how power works to deny, cover-up, or push through COI is important in building the power of public health and nutrition advocates to uncover and resist them in the public interest. The fact that power dynamics work across *levels* from global to local (the final axis of the Power Cube) means that there are also opportunities for like-minded groups to actively form coalitions to counter narratives and actors working against public health interests^13^. Though the power of these coalitions and their interests themselves should also be acknowledged, such a counter-balance to dominant power constellations provides an opportunity to work towards more accountable institutions, better able to deal with emergent COI.

## Addressing Power to Strengthen Action on Conflicts of Interest

In his assessment of global health as a field of power relations (in this journal),^14^ Jeremy Shiffman highlighted three key steps for moving forward: (1) acknowledge COI; (2) analyse power relations; and (3) elevate the place of ‘input legitimacy’ (ie, inclusive deliberation, fair process and transparency). The nutrition community is making some headway in the first, though the Ralston et al paper is an important empirical exposé of strong push-back *against* COI regulation from the types of commercial interests it is designed to keep in check. It also highlights the divergence of interest coalitions on this issue. We argue here and earlier^8^ for stronger attention to the second step, through incorporating power assessment explicitly into COI tools and continuing to research power in international and national nutrition policy processes.

On the third step we have further to go. While COI tools in general aim at fair process, Ralston et al shine a strong light on the politics of the processes that shape the tools in the first place, by noting how WHO member country contributions diverged significantly in some cases (eg, the United States position on any barriers to commercial interests compared to the Colombian position foregrounding public health). It is notable that the SUN Movement (which is also working on its own COI guidelines^[9]^) also reveals something of its own internal politics in two separate and divergent positions submitted to the WHO COI consultation by the Secretariat and by the United Nations Network for SUN. Such positions also reflect the wider geo-politics of public health policy and discourse^15^, including those of the WHO itself and the interest groups which coalesce in the international fora which surround it. We have not yet seen the final WHO COI tool (following this consultation) to be able to judge how WHO has navigated these power processes and incorporated this sharply diverging feedback.

 Ultimately, national governments need to negotiate what are acceptable risks to public health, and the WHO COI tool and others like it are useful for this. We maintain that while the focus on COI is vital, this tool (and others like it) will fail to prevent conflicts of interest unless employed by a robust public sector that is free to weigh decisions in the interests of public health, without fear of influence or compromise from corporate interests. Public and private sectors are more interconnected than ever now – often sharing funding sources, projects and employees – with distinction of authority often quite opaque. As with any system of accountability, the WHO COI tool will fail to operate effectively unless the rules are clearly articulated, including incentives (responsible actors get consulted and included in public health debates) and sanctions (exclusion from policy fora; fiscal and regulatory penalties for more serious practices of corruption and undermining public interest). We would also argue that the ‘informal’ rules of the game need acknowledging, for which power analysis is critical.

 We would now urge the WHO and other public health and nutrition groups to explicitly incorporate the nuanced issue of power into these tools and conversations, for a more rounded and comprehensive view of how COI (both specific and broad) are perpetuated, and how they can be addressed. Having carried out this analysis, civil society, bilateral donors, researchers and international bodies may have a role in supporting countries with lower capacity to design and police such systems, sharing best practice, refining approaches such as the COI tool, and providing technical assistance for monitoring and implementation. Given the key role of bodies such as SUN in providing this kind of assistance, we would recommend that all actors in the nutrition space (not only companies) are held to the same COI standards. And we would welcome more empirical studies, like that of Ralston et al, to further strengthen accountability.

## Ethical issues

 Not applicable.

## Competing interests

 Authors declare that they have no competing interests.

## Authors’ contributions

 Each author contributed materially to the conceptualisation, writing and review of the paper.

## Authors’ affiliations

^1^Institute of Development Studies, University of Sussex, Brighton, UK. ^2^World Vegetable Center, Bangkok, Thailand. ^3^International Food Policy Research Institute, Washington, DC, USA.

## Endnotes


[1] https://scalingupnutrition.org/about-sun/the-sun-movement-strategy/.

[2] https://sdgs.un.org/goals/goal17.

[3] https://www.ifpri.org/blog/principles-practice-and-private-sectors-role-malnutrition-time-review-red-lines.

[4] https://www.who.int/nutrition/consultation-doi/nutrition-tool.pdf?ua=1.

[5] https://www.who.int/nutrition/consultation-doi/nutrition-tool.pdf?ua=1.

[6] https://www.who.int/nutrition/consultation-doi/nutrition-introductory-paper.pdf?ua=1.

[7] https://www.powercube.net/analyse-power/what-is-the-powercube/.

[8] https://ilsi.org/setting-the-record-straight-ilsi-focal-point-in-china-response-to-bmj-and-journal-of-public-health-policy/

[9] https://scalingupnutrition.org/share-learn/multistakeholder-engagement/preventing-and-managing-conflicts-of-interest

